# Prioritization of Biomarker Targets in Human Umbilical Cord Blood: Identification of Proteins in Infant Blood Serving as Validated Biomarkers in Adults

**DOI:** 10.1289/ehp.1104190

**Published:** 2012-01-27

**Authors:** Nicole Hansmeier, Tzu-Chiao Chao, Lynn R. Goldman, Frank R. Witter, Rolf U. Halden

**Affiliations:** 1 Swette Center for Environmental Biotechnology, Biodesign Institute, Arizona State University, Tempe, Arizona, USA; 2 Department of Chemistry and Biochemistry, Arizona State University, Tempe, Arizona, USA; 3 School of Public Health and Health Services, George Washington University, Washington, District of Columbia, USA; 4 Department of Gynecology and Obstetrics, Johns Hopkins University School of Medicine, Baltimore, Maryland, USA; 5 Department of Environmental Health Sciences, Johns Hopkins Bloomberg School of Public Health, Baltimore, Maryland, USA; 6 Center for Health Information and Research, Arizona State University, Tempe, Arizona, USA

**Keywords:** body fluid, diagnostics, disease, LC-MALDI-MS, pathways, proteomics

## Abstract

Background: Early diagnosis represents one of the best lines of defense in the fight against a wide array of human diseases. Umbilical cord blood (UCB) is one of the first easily available diagnostic biofluids and can inform about the health status of newborns. However, compared with adult blood, its diagnostic potential remains largely untapped.

Objectives: Our goal was to accelerate biomarker research on UCB by exploring its detectable protein content and providing a priority list of potential biomarkers based on known proteins involved in disease pathways.

Methods: We explored cord blood serum proteins by profiling a UCB pool of 12 neonates with different backgrounds using a combination of isoelectric focusing and liquid chromatography coupled with matrix-assisted laser desorption/ionization tandem mass spectrometry (MALDI-MS/MS) and by comparing results with information contained in metabolic and disease databases available for adult blood.

Results: A total of 1,210 UCB proteins were identified with a protein-level false discovery rate of ~ 5% as estimated by naïve target-decoy and MAYU approaches, signifying a 6-fold increase in the number of UCB proteins described to date. Identified proteins correspond to 138 different metabolic and disease pathways and provide a platform of mechanistically linked biomarker candidates for tracking disruptions in cellular processes. Moreover, among the identified proteins, 38 were found to be approved biomarkers for adult blood.

Conclusions: The results of this study advance current knowledge of the human cord blood serum proteome. They showcase the potential of UCB as a diagnostic medium for assessing infant health by detection and identification of candidate biomarkers for known disease pathways using a global, nontargeted approach. These biomarkers may inform about mechanisms of exposure–disease relationships. Furthermore, biomarkers approved by the U.S. Food and Drug Administration for screening in adult blood were detected in UCB and represent high-priority targets for immediate validation.

Many diseases are of early-life origin. Early diagnosis of diseases, toxic exposures, effects, and susceptibilities in the still-developing body of infants will be required to develop successful intervention and treatment strategies to battle diseases. It is well documented that exposures to environmental chemical contaminants, including cigarette smoke constituents, for example, have adverse effects on fetal development and result in unfavorable health trajectories for affected children (Apelberg et al. 2007a; Buczyńska and Tarkowski 2005; Mattison 2010; Miranda et al. 2009; Wigle et al. 2007). Long-term outcomes such as diabetes, obesity, and chronic heart and kidney diseases have all been hypothesized or postulated to have their basis in fetal and childhood exposure (Barker et al. 2002) and show an increased prevalence in children and newborns (Bloomgarden 2004; Ferrara 2007). Therefore, the development of early diagnostics as predictors for child health is of paramount importance to enable early intervention.

Umbilical cord blood (UCB) is a very attractive biological specimen, because relatively high volumes (up to tens of milliliters) of this biofluid are available for sampling without posing an added risk and burden to the newborn or its mother in the process. In addition, it has the potential to inform about existing or potential future adverse effects. UCB is already in use for prediagnosis and treatment of immune deficiencies (Notarangelo 2010). However, its primary use at this time is for bone marrow transplantation (Buchheiser et al. 2009).

Mass spectrometry (MS)–based proteomics is a powerful technology, allowing for the identification and quantification of hundreds of proteins in parallel from a single sample without necessitating prior selection or exclusion of potential analytical targets. It has been employed successfully for a diverse range of organisms, tissues, and biofluids (Aebersold and Mann 2003; Ahrens et al. 2010a, 2010b; Beck et al. 2011; Domon and Aebersold 2010; Nilsson et al. 2010) and offers an ideal platform for fast identification of new protein markers for diseases or adverse exposure (Lemos et al. 2010; Liumbruno et al. 2010). For adult blood, there is a long tradition of analyzing serum proteins using two-dimensional gel electrophoresis (Hughes et al. 1992) or shotgun proteomics (Omenn et al. 2005; Pieper et al. 2003; Richter et al. 1999; Schenk et al. 2008; States et al. 2006), with continuous analytical improvements (Bell et al. 2009; Gaso-Sokac and Josic 2010; Rai et al. 2005). In contrast to adult blood, much less information is available for the cord blood proteome. Recently, two short overviews on cord blood proteomics were published describing 207 and 837 different proteins, respectively (Colquhoun et al. 2009; Song et al. 2009). Unfortunately, the latter report provided information on only 61 of the 837 proteins. The identifiers of the 776 remaining proteins are not published.

More detailed investigations of the UCB proteome are needed to accelerate the pace of discovery and expand the spectrum of infant health diagnostics. In addition, the sampling of UCB for proteome studies has to be successful within the limitations of clinical reality and is therefore much more challenging than the analysis of adult blood. The aim of this study was to provide insights into several important aspects necessary to use the UBC as an effective source for protein-based biomarkers. As a first priority, we aimed to expand the knowledge on the detectable proteome in UCB, which can be obtained from a limited starting volume. This is especially important because large volumes of UCB are required to obtain sufficient stem cells for therapeutic purposes (Buchheiser et al. 2009; Forraz and McGuckin 2011). Hence, reducing the required volume for diagnostic purposes to a minimum is highly desirable. Moreover, we compared the identified proteins with known and proposed biomarkers to provide a short list of potential biomarkers that form a basis for the exploration of molecular mechanisms of exposure-disease relationships.

## Materials and Methods

*Chemicals.* All chemicals were obtained from Sigma-Aldrich (Sigma Aldrich, St. Louis, MO, USA) with the following exceptions: Sequencing-grade modified porcine trypsin was obtained from Promega (Madison, WI, USA), and Bradford reagent was purchased from Bio-Rad (Hercules, CA, USA).

*UCB serum samples.* The UCB serum samples were acquired from the cord blood cohort collection of the Baltimore Tracking Health-Related Environmental Exposures (THREE) study. This study was approved by the Johns Hopkins Medicine Institutional Review Board (IRB approval 04-04-22-02) and received a waiver from the Health Insurance Portability and Accountability Act (2002). The study showed U.S.-representative exposure levels (mean and maximum concentrations) of *a*) perfluorooctanoate (PFOA): 1.6 ng/mL, 7.1 ng/mL; *b*) perfluorooctane sulfonate (PFOS): 5 ng/mL, 34.8 ng/mL; *c*) organochlorine pesticides (e.g., *trans*-nonachlor): 94 pg/mL, 185.5 pg/mL; *d*) *trans*-permethrin: 36.3 pg/mL, 34.8 ng/mL; *e*) polychlorinated biphenyls (PCBs) (e.g., di-*ortho*): 17.4 ng/g lipid, 176.5 ng/g lipid; and *f*) heavy metals (e.g., lead: 0.66 μg/dL, 15.5 μg/dL; copper: 38.6 μg/dL, 265 μg/dL). Details on this cohort are described elsewhere (Apelberg et al. 2007a, 2007b; Herbstman et al. 2007; Neta et al.2011; Wells et al. 2011). Of this cohort study, 12 samples (eight male and four female) (Table 1) were randomly chosen for proteomic profiling. Using the Witter cord cradle (Witter et al. 2001), hospital-trained personnel collected the UCB by direct venipuncture of the umbilical vein, which assured that no maternal blood was present in the samples. In addition, analysis of the X:Y chromosome ratios in male newborns as described by Guerrero-Preston et al. (2010) further confirmed the absence of maternal blood. Up to five 10-mL UCB samples were collected per newborn and immediately stored at 4°C. Within < 3 hr, the refrigerated blood specimens were centrifuged at 1,000 × *g* for 15 min to collect the serum. Serum samples were then fractionated and stored in 2-mL polypropylene cryovials at –80°C. For proteomic analysis, frozen sample splits were shipped on dry ice to the Biodesign Institute at Arizona State University; the individual samples were thawed for the first time just before sample processing.

**Table 1:** Statistics of selected UCB donors.

Characteristics	No. of donors
Maternal race	
African American	6
Caucasian	5
Asian	1
Maternal education	
≤ High school diploma	7
1–4 years of college	2
≥ 5 years of college	3
Health insurance status	
Private	5
Medicaid	3
Uninsured	4
Body mass index	
Overweight	1
Obese	3
Substance abuse	
Smoking	2
Reported diseases during pregnancy	
Anemia	1
Asthma	1
Thyroid condition	1
Sexually transmitted disease	1
Urinary tract infection	1
Maternal age (years)	17–36
Gestational age (days)	270–288


*Preparation of reference pool samples and immunodepletion.* Aliquots of 100 μL of each of the 12 individual UCB samples were pooled to obtain a composite sample with a protein concentration of 79 mg/mL protein as determined by Bradford assay. Of this pool, 240 μL were taken and human serum albumin (HSA) depleted using a Vivapure anti-HSA kit (VivaScience, Hannover, Germany) according to the manufacturer’s description. We chose not to further deplete the samples because several of the other highly abundant blood proteins often routinely depleted are either U.S. Food and Drug Administration (FDA)–approved adult blood biomarkers (Anderson 2010) or proposed biomarker candidates for diverse diseases or exposures (Colquhoun et al. 2009; Ehmann et al. 2007; Ward et al. 2006). The HSA-depleted proteome fraction was then concentrated and desalted by ultrafiltration using the Vivaspin 500 concentrators (MWCO 3kDa; Sartorius, Goettingen, Germany). The total volume of the resultant composite sample was 150 μL, with a protein concentration of 33 mg/mL.

*Protein digest and sample fractionation.* Proteins were denatured and reduced in 10 mM ammonium bicarbonate and 0.05% sodium dodecyl sulfate with 10 mM dithiothreitol at room temperature for 1 hr. Alkylation of proteins was accomplished by incubation in 40 mM iodoacetamide for 1 hr at room temperature in the dark. Ten microliters sequencing-grade modified porcine trypsine (1 mg/mL stock solution; Promega, Madison, WI, USA) was added, and the mixture was incubated at 37°C overnight. Tryptic digests (100–500 μg) were then fractionated with the Agilent 3100 OFFGEL Fractionator using the 3100 OFFGEL Low Res Kit, pH 3-10 (Agilent Technologies, Santa Clara, CA, USA). The isoelectric focusing (IEF) was performed without ampholytes and glycerol according to the manufacturer’s instructions for peptide focusing. In short, the peptides were separated in a linear gradient of up to 8,000 V. The potential was kept at 8,000 V until 56,000 Vh was reached. The 12 IEF fractions were then extracted with 0.1% trifluoroacetic acid in 50% methanol, vacuum-concentrated, and dissolved in 2% acetonitrile and 0.1% trifluoroacetic acid.

*Reverse-phase liquid chromatography (RP-LC) separation and MS-analysis.* For the RP nano-LC separation, a Tempo LC MALDI Spotting system (Applied Biosystems/MDS SCIEX, Foster City, CA, USA) was used with a 2-μL injector loop and a Chromolith CapRod column (150 × 0.1 mm; Merck, Darmstadt, Germany). Separation was obtained by running a gradient at a 2-μL/min flow rate. Solution A contained 2% LC-grade acetonitrile and 0.1% trifluoroacetic acid; solution B contained 98% acetonitrile and 0.1% trifluoroacetic acid. A 30-min gradient elution with the following parameters was used: 2% B (0.5 min), 2%→40% B (0.5–15 min), 40%→65% B (15–22 min), 65%→80% B (22–24 min), 80% B (24–26 min), 80%→2% B (26–28 min), 2% B (28–30 min). The matrix-assisted laser desorption/ionization (MALDI) matrix solution (7 mg/mL recrystallized α-cyano-hydroxycinnamic acid, 0.1% trifluoroacetic acid, 70% acetonitrile) was added postcolumn with a flow rate of 2 μL/min. Every 7 sec the combined eluate was automatically spotted onto a matrix prespotted stainless steel MALDI target plate (Applied Biosystems/MDS SCIEX). For calibration, 13 calibrant spots (ABI 4700 Mix) were added to each plate manually. All spotted samples were analyzed with a 4800 MALDI-tandem time-of-flight (TOF/TOF) mass spectrometer (Applied Biosystems/MDS SCIEX). First, MALDI-MS spectra were acquired over a mass range of *m/*z 800–4,000 in positive-ion reflector mode using 70–500 laser shots/spectrum with a fixed relative laser power of 3,300 and a central biased spot search pattern. In each MS spectrum, up to 25 peaks were selected for MS/MS using an acquisition method that excluded ions with signal-to-noise (S/N) ratios of < 50. The precursor ion with the weakest S/N ratio was acquired first to achieve the maximum signal intensity for low-abundance peptides. Tandem MS mode was operated using air as the collision-induced dissociation gas and enabled metastable ion suppressor settings. The relative precursor mass window was set to 200 (full width half mass). The MS/MS acquisition of selected precursors was set to a maximum of 2,500 shots/spectrum with a fixed relative laser power of 4,200.

*Protein identification and pathway mapping.* The combined MS/MS spectra were searched using ProteinPilot™ Software v3.0 (version 3.01 prior to July 2009; Applied Biosystems/MDS SCIEX) with the implemented Paragon and the Pro Group processing algorithm against the human subset of the National Center for Biotechnology Information (Bethesda, MD, USA) nonredundant protein database (downloaded on 7 January 2008) and the UniProt human proteome database (version from 20 March 2009). Peptide and protein identification was carried out with ProteinPilot^TM^. Most search parameters are not user-adjustable but confer to the molecular and cellular proteomics guidelines (Carr et al. 2004). Adjustable search parameters included cysteine modification by iodoacetamide, methionine oxidation, tryptic digestion, and thorough search with biological modifications ID focus. Additional information on the ProteinPilot^TM^ algorithm is found in Shilov et al. (2007). Because the samples were obtained according to normal clinical procedures and settings, they may include proteins that were at least partially digested, proteolysed, or degraded during sample handling (Richter et al. 1999). To address this issue and also to identify partially digested proteins, additional database searches were performed allowing nonspecific digestion as search parameter. This search strategy was successfully employed for the generation of a plasma proteome reference map (Adkins et al. 2002; Chan et al. 2004; Richter et al. 1999). Protein identification was based on ProtScore unused score criteria (Pro Group Algorithm, ProteinPilot^TM^ software; Applied Biosystems/MDS SCIEX). Only proteins identified with Protscore ≥ 1.3 and at least one unique peptide with ≥ 95% confidence were used for further analysis. False discovery rate (FDR) was estimated by a search against a randomized decoy database using the same parameters as the original search (Elias et al. 2005) as well as using the MAYU approach (Reiter et al. 2009).

Blast2GO software was used to extract gene ontology (GO) information for each protein (Götz et al. 2008) for functional characterization. The resulting list was curated manually. Pathway information for the identified proteins was obtained by Basic Local Alignment Search Tool (BLAST searches of the proteins against the curated Kyoto Encyclopedia of Genes and Genomes (KEGG) database (Aoki-Kinoshita and Kanehisa 2007).

*Supporting information.* Beyond the results presented here and in the Supplemental Material (http://dx.doi.org/10.1289/ehp.1104190), the additional information (EHP_Appendix 1–4 and the spectral data folder) is available on our homepage http://labs.biodesign.asu.edu/halden/publications/ (Hansmeier et al. 2012) and at http://proteomecommons.org (ProteomeCommons.org 2012) project UCB proteome. Appendix 1 contains the extended version of the list of all identified UCB proteins and their descriptions. Appendix 2 lists those proteins that are shared between the UCB proteome and the proteome reported for adult blood. Appendix 3 lists UCB proteins assigned to GO category “multicellular organismal development” which are unique for UCB or shared with adult blood. Appendix 4 shows a detailed overview of UCB proteins and their respective KEGG pathways involved.

## Results

An important step forward for the evaluation of the diagnostic potential of the UCB proteome as a source of biomarkers is the comprehensive analysis of its protein composition, ideally with minimal bias from individual health histories. Therefore, we created pooled UCB composite samples from the THREE cohort study, which showed U.S.-representative exposure levels to a diverse range of environmental pollutants, including PFOS, PFOA, organochlorine pesticides, permethrin, and PCBs, as well as heavy metals (see “Materials and Methods” and Apelberg et al. 2007b; Herbstman et al. 2007; Neta et al. 2011; Wells et al. 2011). Using a total of 240 μL of the composite sample as starting material, we acquired a total of 62,686 MS/MS spectra from 12 different LC-MALDI-TOF/TOF runs. Altogether 1,210 nonredundant human proteins were identified with ≥ 95% confidence [see Supplemental Material, Table 1 (http://dx.doi.org/10.1289/ehp.1104190), and Appendix 1, Hansmeier et al. 2012]. The associated FDR were estimated to be 5.1% using a naïve target-decoy search and 0.049 [~ 0.002 PSM (peptide spectrum match) FDR] using MAYU. A subset of 843 proteins was identified at the ≥ 99% confidence level (~ 0.009 protein FDR, ~ 0.0005 PSM FDR, MAYU).

**Figure 1:**
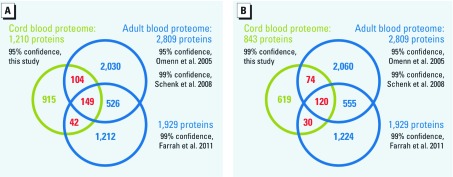
Comparison of the 95% (*A*) and 99% (*B*) confidence UCB proteome data sets (this study) with data sets generated from individual adult blood proteome studies (Omenn et al. 2005; Schenk et al. 2008) and a meta-analysis of high-quality data sets from the last 6 years (Farrah et al. 2011).

Compared with the previously published UCB proteome (Colquhoun et al. 2009) generated using the same search algorithm and confidence cutoff at ≥ 95%, our data set represents an approximately 6-fold increase in the number of identified UCB proteins to date. We further compared the UCB to a nonredundant list of plasma proteins of adults using published data (Omenn et al. 2005; Schenk et al. 2008). In addition, we compared our data with a recently published high-quality data set obtained by the meta-analysis of 91 experiments (Farrah et al. 2011). In total we found 295 proteins shared between the UCB and adult proteomes (Figure 1A; see also Appendix 2, Hansmeier et al. 2012). The number of shared proteins is reduced slightly to 224 when only UCB proteins identified with 99% confidence are considered (Figure 1B). Thus, a subgroup of blood proteins exists that can be consistently identified regardless of the methods and samples used.

The detected UCB proteome covered a large range of molecular weights (4.7–3,880 kDa, as calculated from the sequence of the proteins). About 44.6% of the detected proteins are classified as extracellular [see Supplemental Material, Figure 1 (http://dx.doi.org/10.1289/ehp.1104190)]. Further, 33.3% and 22.1% of the identified proteins were predicted to be intracellular or membrane UCB proteins, respectively. Similar distributions of extracellular, intracellular, and membrane proteins were observed in adult blood of healthy donors (Schenk et al. 2008). The presence of these proteins in blood is generally believed to be the result of cell lyses, tissue leakage or shedding from cell surfaces, and subsequent releases in the bloodstream (Adkins et al. 2002; Chan et al. 2004; Schenk et al. 2008).

**Figure 2:**
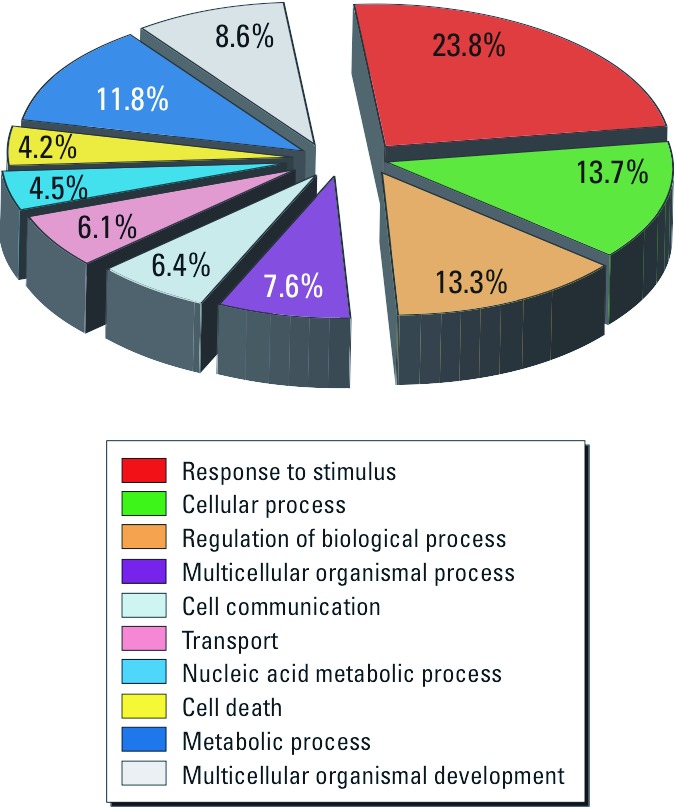
Classification of the UCB proteome according to GO.

In general, protein biomarkers are most useful if their functions are known and thus can be mechanically linked to the molecular basis of adverse health effects. Functional assignments of the identified UCB proteins according to GO classification (Figure 2) revealed that a plurality of UCB proteins (23.8%) were involved in responses to different stimuli in particular immune responses (e.g., immunoglobulins, signal transduction proteins, and elements of the complement system). Furthermore, a large number of proteins were involved in cellular (13.7%), regulatory biological (13.3%), and metabolic processes (11.8%). Several of these proteins are known to be actively secreted into the bloodstream—for example, to maintain homeostasis in the body. An example is angiotensinogen, which is part of the renin–angiotensin system and regulates blood pressure. An interesting category, especially with respect to child development, is the GO class of multicellular organismal development, describing several critical proteins involved in embryonic skeletal/bone system development essential for fetal growth (see Appendix 3, Hansmeier et al. 2012). About 8.6% of identified proteins belonged to this group, including homeobox protein DLX-6 (P56179), sickle tail protein homolog (Q5T5P2), and fetuin-A (P02765) (Graham 2002; Karamessinis et al. 2008; Semba et al. 2006). Although these proteins are not necessarily specific to the UCB proteome, their importance for embryogenesis is well recognized.

**Figure 3:**
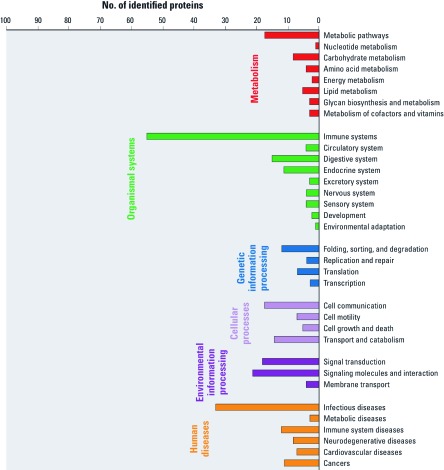
Overview of UCB proteins mapped to KEGG pathways. See Appendix 4 (Hansmeier 2012) for more detailed information.

Moreover, we organized the UCB proteome into metabolic pathways according to the KEGG database to identify which cellular processes may be traced using UCB. A total of 138 metabolic and signaling pathways were found to be active (Figure 3; see also Appendix 4, Hansmeier et al. 2012). This includes proteins involved in immune response pathways, in control of the circulatory, endocrine, digestive, nervous systems, and in cellular metabolism and catabolism. Furthermore, traceable proteins belonged to a diverse range of signaling pathways in developmental processes, for example, for calcium, MAPK (mitogen-activated protein kinases), WNT (signaling pathway first discovered in *Drosophila melanogaster*), and Jak-STAT (Janus kinase-signal transducer and activator of transcription). Moreover, 54 proteins were already assigned to known human disease pathways and might be relevant for tracking disease-related changes earlier in life associated with maturity-onset diabetes of the young (MODY), primary immunodeficiency, systemic lupus erythematosus, ventricular/hypertonic or dilated cardiomyopathy, diverse kind of cancers, or infectious diseases.

Finally, we identified proteins in UCB that already are approved as biomarkers in adults by the FDA [Supplemental Material, Table 2 (http://dx.doi.org/10.1289/ehp.1104190)], including 38 proteins diagnostic of a diverse range of disorders, for example, artery and organ diseases and inflammation in adults (Anderson 2010).

## Discussion

The rising prevalence of newborn diseases with long-term effects (Bloomgarden 2004; Ferrara 2007), such as diabetes, obesity, and chronic heart and kidney diseases, is expected to usher in new methods of UCB screening for early diagnosis in neonates. The present data set represents an important step forward in characterizing the proteome UCB composition and detecting candidate biomarkers therein. The high-confidence UCB data set (≥ 99% confidence, 1% protein FDR) of the UCB proteome is comparable in size with the high-quality data set from Schenk et al. (2008). As expected, a significant portion (~ 25%) of the proteins detected in UCB was previously found in adult blood. Because only limited information is available regarding the concentration range of UCB proteins, we estimated the depth of our analysis from known adult blood protein levels. For instance, among identified targets were titin and mitochondrial glycine amidinotransferase, two proteins known to occur in the low nanogram per milliliter range (4–6.8 ng/mL) in blood (Farrah et al. 2011). Assuming that proteins occur at comparable levels in adult and fetal blood (i.e., within an order of magnitude), our data likely covered a dynamic range of about six to seven orders of magnitude, which is in line with prior reports on adult blood (e.g., Chan et al. 2004).

We demonstrated that as little as 240 μL cord blood serum is suitable for obtaining proteome information at confidence levels comparable with those attained in adult blood studies. Use of this relatively low sample volume is not anticipated to adversely affect the possibility of further diagnostic tests.

Because clinical approval and validation of biomarkers are costly and time consuming (Chao et al. 2010), prioritization is important. Of highest priority are UCB proteins already approved by the FDA for diagnosis in adults. Among these were protein biomarkers for different organ diseases such as deficiency of thyroid binding (thyroxine-binding globulin), chronic kidney dysfunctions (cystatin C), hypercoagulation disorders (plasminogen), and cardiovascular diseases (apolipoprotein A1, B). In addition, deficiencies in coagulation factors X or VII may indicate bleeding disorders that, without proper therapy, can lead to liver failure, internal bleeding, or sudden death (Morley 2011). It is conceivable that these established biomarkers are also of diagnostic value in infants.

Second in importance are proteins with known functions in metabolic pathways to inform about disruptions of normal cellular processes. Although not approved by the FDA for diagnostic purposes, a number of the proteins identified in the UCB proteome are either known to play a role in certain diseases or can be diagnostic of exposure to toxic substances. Among the proteins found in disease pathways is the cardiac ryanodine receptor 2, whose dysfunction is directly linked to cardiac arrhythmia and sudden heart failure (Betzenhauser and Marks 2010; Durham et al. 2007; Wehrens and Marks 2003). Also of interest are homeobox protein NKX6.1 for MODY (Donelan et al. 2010); protein S and platelet-derived growth factor BB for acute kidney injury (Thurman et al. 2009); WNT target-gene promoter TBL1XR1 (F-box-like/WD repeat containing protein) for acute lymphoblastic leukemia (Parker et al. 2008); and proto-oncogene serine/threonine-protein kinase PIM1, which is proposed as a marker for mutated K-*ras* signaling activity in pancreatic cancer (Xu et al. 2011). Furthermore, peroxisome proliferator-activated receptor (PPAR) and calcium-signaling pathways are associated with metabolic diseases such as diabetes mellitus type 2 and cardiovascular disorders (Benkusky et al. 2007; Biscetti et al. 2009; Bulhak et al. 2009). Platelet factor 4, pro-platelet basic protein precursor, and complement component 3 are known to be diagnostic in a panel for acute lymphoblastic leukemia in children (Shi et al. 2009). Interestingly, lower adiponectin levels in UCB also have been found to be a predictor of adiposity in children at 3 years of age (Mantzoros et al. 2009).

We also detected several proteins associated with different kinds of toxic exposures. Toxic exposures can often have varied unspecific effects on human health. Accordingly, their effects on the molecular level are largely unknown. Potential biomarkers of exposure identified in here include proteins involved in lipid metabolism disruptable by smoking (Craig et al. 1989), alcohol (Través et al. 2007), or bisphenol A exposure (Chou et al. 2011). Specifically, we detected all previously proposed protein biomarkers of *in utero* exposure to tobacco products from maternal smoking (Colquhoun et al. 2009).

Other identified proteins of interest included alpha-fetoprotein, whose expression is affected by cigarette smoke exposure but has also been found to be elevated after exposure to dioxins and phenols (Colquhoun et al. 2009; El Far et al. 2006), and endothelin B, which is elevated after inhalation of diesel exhaust (Langrish et al. 2009; Peretz et al. 2008). Antenatal administration of the drug betamethasone was associated with an increase in UCB of retinol-binding protein, transthyretin, and transferrin (Georgieff et al. 1988). Increased levels of IgG were found to be associated with a range of environmental exposures—for example, to mold (Rydjord et al. 2007), organochlorines (Karmaus et al. 2005), tobacco-smoke products (Colquhoun et al. 2009), and methylmercury (Nyland et al. 2011).

Overall, the presented data indicate that proteome analyses may provide a window to current physiological status of patients and also offer opportunities for detecting molecular malfunctions related to diseases. In particular, protein biomarkers with mechanistic linkage to disturbed signaling and metabolic pathways have proved to be of diagnostic value. Diagnostics in the form of protein assays provide a spectrum of clinical data, including information on acute events such as forecasting of coronary diseases, myocardial infarction, and cancer (Anderson 2010).

Ideally, the results of this study will be supplemented with quantitative information on a population basis. Knowledge of normal and aberrant protein abundance at the proteome level, together with pathway-centered analyses of adverse health effects in diverse population, will continue to drive the development of mechanistically based biomarkers of health status.

## Conclusion

In this study, we furnish a comprehensive data set of proteins detectable in UCB by MS. Here, detected proteins shown previously to be of diagnostic value in adults are deemed to be of particular interest as biomarker candidates. This UCB proteome screen demonstrates the feasibility of viewing numerous indicators of health effects and diseases in infants, using a global approach. The data set obtained may serve as a platform for further targeted and quantitative analyses of UCB proteins.

## Supplemental Material

(336 KB) PDF
