# Supplementary material for: Prioritization of Biomarker Targets in Human Umbilical Cord Blood: Identification of Proteins in Infant Blood Serving as Validated Biomarkers in Adults
Source: Environ Health Perspect. 2012 Jan 27;120(5):764–9. doi: 10.1289/ehp.1104190 (PMC3346780; doi:10.1289/ehp.1104190)
Supplement: (336 KB) PDF [file ehp.1104190.s001.pdf]

Supplementary Material

Prioritization of Biomarker Targets in Human Umbilical Cord Blood: Identification of Proteins in Infant Blood Serving as Validated Biomarkers in Adults

Nicole Hansmeier, Tzu-Chiao Chao, Lynn R. Goldman, Frank R. Witter and Rolf U. Halden

Table of contents:

1. Supplemental Material, Figure 1: p. 2
2. Supplemental Material, Table 1: p. 3-7
3. Supplemental Material, Table 2: p. 8
4. Reference: p. 9

Additional information is available on our homepage
http://labs.biodesign.asu.edu/halden/publications/
N. Hansmeier, T.C. Chao, L.R. Goldman, F.R. Witter and R.U. Halden (2012) Prioritization of Biomarker Targets in Human Umbilical Cord Blood: Identification of Proteins in Infant Blood Serving as Validated Biomarkers in Adults. Environ. Health Perspect.
Full text file contains EHP Appendix 1-4 and MS data files
and at http://proteomecommons.org, project: UCB proteome.

A

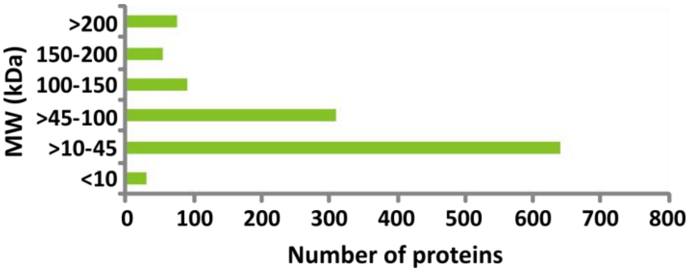

B

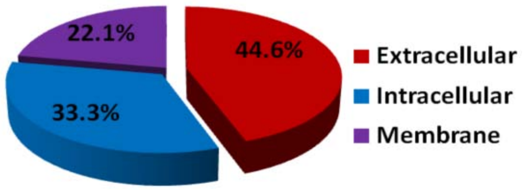

Supplemental Material, Figure 1. Characteristics of the UCB proteome.

A Histogram showing the molecular weight distribution of the calculated masses of the 1,210 observed UCB proteins calculated with the sequence manipulation suite.

B Pie chart showing the classification of UCB proteins according to GO cellular components.

Supplemental Material, Table 1. Abbreviated list of the 1,210 identified UCB proteins (extended version is available on our homepage http://lab.biodesign.asu.edu/halden/publications & at http://proteomecommons.org).

N	Accession (GI & Uniprot)		N	Accession (GI & Uniprot)		N	Accession (GI & Uniprot)		N	Accession (GI & Uniprot)		N	Accession (GI & Uniprot)		N		
1	gi|4502027	P02768	46	gi|28810	P02749	91	gi|62897915	Q53FV4	136	gi|77379458		181	gi|21669653		226	gi|62001846	
2	gi|66932947		47	gi|119585668	B7Z545	92	gi|63102951		137	gi|90994746		182	gi|62089402	Q59EA3	227	gi|63102995	
3	gi|112911	P01023	48	gi|88853069	P04004	93	gi|21707947	Q68CK4	138	gi|18025702		183	gi|84798010		228	gi|8101647	
4	gi|115298678	P01024	49	gi|158254550	A8K3E4	94	gi|78191798	P03952	139	gi|12655720		184	gi|58222928		229	gi|98956182	
5	gi|4557871	P02787	50	gi|189083780	P06396	95	gi|22595063		140	gi|21669979		185	gi|8118083	O75882	230	gi|58222902	
6	gi|27370813		51	gi|34535785		96	gi|24430192	P08779	141	gi|34526063	P27169	186	gi|21669649		231	gi|21669331	
7	gi|15990507	P01009	52	gi|1197209	P02763	97	gi|70798701		142	gi|408299	P02656	187	gi|170684604		232	gi|112701473	
8	gi|178730	C0JYY2	53	gi|4504349	D9YZU5	98	gi|106898223		143	gi|4557323	Q6IB74	188	gi|2077999	Q65ZC9	233	gi|119596170	
9	gi|2347136	A2BHY4	54	gi|33451	P01871	99	gi|4502163	P05090	144	gi|48145977		189	gi|114147285		234	gi|50897468	
10	gi|47132555	P02751	55	gi|112877	P02763.1	100	gi|122892088		145	gi|62897073	P15814	190	gi|64654564	Q4VAI6	235	gi|41019454	
11	gi|77744385	P08603	56	gi|189066632	B2R8I2	101	gi|24899162	Q8IZZ5	146	gi|871345		191	gi|118405803		236	gi|15637449	
12	gi|34364820		57	gi|21669631		102	gi|158258685	A8K9A9	147	gi|95101768		192	gi|112694740		237	gi|4321595	
13	gi|158255874	A8K5A4	58	gi|50659080	P01011	103	gi|114147558		148	gi|21669497	Q8WZ42	193	gi|109693178		238	gi|34364639	Q6N095
14	gi|149673887		59	gi|18044959		104	gi|215982736		149	gi|17066105		194	gi|109693188		239	gi|3413516	O75636
15	gi|119599289	P00450	60	gi|21669449		105	gi|48145933	P02766	150	gi|119593796		195	gi|31076441		240	gi|33319550	
16	gi|4557321	P02647	61	gi|579676	P02760	106	gi|58372399	Q5EK51	151	gi|33319616		196	gi|47846547		241	gi|4378370	
17	gi|455970	P02774	62	gi|21669327		107	gi|39652254	Q06033	152	gi|114147592	Q8NFP9	197	gi|21757089		242	gi|62204995	
18	gi|189053897		63	gi|194375081	B4DVE1	108	gi|106897586		153	gi|62422577		198	gi|4502503	P04003	243	gi|58221960	
19	gi|11321561	P02790	64	gi|73858568	P05155	109	gi|2921526		154	gi|84798412		199	gi|89191868	P04275	244	gi|41019528	Q9Y6V0
20	gi|70778918	P19823	65	gi|119395750	P04264	110	gi|20178323	P36955	155	gi|112697187	P13645	200	gi|122892362		245	gi|106897477	
21	gi|4505881	P00747	66	gi|73858566	P05546	111	gi|93007626		156	gi|195972866	B0YIW2	201	gi|109240688		246	gi|150378498	Q2LD37
22	gi|27552515		67	gi|37181420	Q96PD5	112	gi|119763	P00748	157	gi|167887493	A8K5J8	202	gi|170684522		247	gi|13016667	
23	gi|156231037	P01042	68	gi|12054078		113	gi|28317		158	gi|158256062	P05160	203	gi|47940520	Q6IN99	248	gi|6643619	
24	gi|67624831	P00734	69	gi|21669435		114	gi|169205253		159	gi|158254552		204	gi|112696621		249	gi|8250281	
25	gi|189054564	P02771	70	gi|119573007	P02652	115	gi|292221		160	gi|33319482		205	gi|119623150	P05543	250	gi|4758502	Q14520
26	gi|6715607	P69892	71	gi|62089238		116	gi|6492197		161	gi|18025668	Q6ZP87	206	gi|62898910	D3DNU8	251	gi|112702678	
27	gi|4501987	P43652	72	gi|189054178		117	gi|62896541	Q53HT9	162	gi|34526391	Q96L91	207	gi|77379426		252	gi|34530413	Q6UXB8
28	gi|93163358	P06727	73	gi|42716297	P10909	118	gi|51173528	P22792	163	gi|56549696	P04053	208	gi|114386092		253	gi|3337391	P00739
29	gi|156523970	P02765	74	gi|62089410	Q59E99	119	gi|75707225		164	gi|63054852		209	gi|119626079		254	gi|4323912	
30	gi|34365089	Q6MZX7	75	gi|21669425		120	gi|8777875		165	gi|481341		210	gi|119626104		255	gi|32480766	Q149N8
31	gi|194376340		76	gi|34534305		121	gi|194388850	B4DRY0	166	gi|33319552		211	gi|12054074		256	gi|112696203	
32	gi|189053442	B2R6W1	77	gi|215982760		122	gi|4502495	P09871	167	gi|95007517	B2R5G8	212	gi|121488433		257	gi|2058536	
33	gi|45580688	P10643	78	gi|119631418	D3DPG0	123	gi|21669671		168	gi|189053309	B2R5G8	213	gi|130381779		258	gi|34527235	
34	gi|119610986	P08697	79	gi|62861016		124	gi|118406110		169	gi|18496799		214	gi|156627579	P05452	259	gi|156076800	
35	gi|189069199	P01008	80	gi|194387702	B4DRF2	125	gi|587420		170	gi|87299000		215	gi|183763	Q03591	260	gi|11118905	
36	gi|194384366	B4E1Z4	81	gi|17939658		126	gi|1200069		171	gi|4757760	Q15848	216	gi|197115843		261	gi|45935385	O94885
37	gi|46577680	P04217	82	gi|38488662	P13671	127	gi|14289024	O95445	172	gi|156633078		217	gi|21669323		262	gi|28590	
38	gi|33989		83	gi|158256710	P07996	128	gi|6841322		173	gi|119609221		218	gi|218512079	P01859	263	gi|29725624	Q86Y22
39	gi|178853	P02649	84	gi|4262117		129	gi|3327990		174	gi|112701253		219	gi|33126535	Q7Z5F4	264	gi|18092618	
40	gi|62089124	Q59EP2	85	gi|38016947	P01031	130	gi|21669651		175	gi|1071953		220	gi|4502157	P02654	265	gi|2687853	Q92878
41	gi|156119625	P19827	86	gi|55743122	P02753	131	gi|284238		176	gi|39794308		221	gi|4503107	P01034	266	gi|3091192	
42	gi|4503689	P02671	87	gi|80975643		132	gi|112694411		177	gi|119617033		222	gi|4586460	Q8NHU6	267	gi|74146374	
43	gi|1483187	Q14624	88	gi|55663330	Q5VY30	133	gi|119598461		178	gi|170684498		223	gi|46253810		268	gi|215274225	Q5VST9
44	gi|13650074	P69905	89	gi|3540267		134	gi|21669073		179	gi|28375497	P29622	224	gi|58221677		269	gi|30841934	
45	gi|141596	P25311	90	gi|106575		135	gi|75707613		180	gi|86793109	P17927	225	gi|58223260		270	gi|58202736	

<table>
<tr><td>271</td><td>gi|6855601</td><td></td><td>320</td><td>gi|58222078</td><td></td><td>369</td><td>gi|71891693</td><td>P98196</td><td>418</td><td>gi|791049</td><td>P50440</td><td>467</td><td>gi|119626065</td><td></td><td>516</td><td>gi|119627049</td><td>P07357</td></tr>
<tr><td>272</td><td>gi|56550039</td><td>Q03164</td><td>321</td><td>gi|62420884</td><td>Q16832</td><td>370</td><td>gi|217272887</td><td>Q8IWG1</td><td>419</td><td>gi|169164581</td><td></td><td>468</td><td>gi|1407576</td><td></td><td>517</td><td>gi|119627051</td><td></td></tr>
<tr><td>273</td><td>gi|681900</td><td></td><td>322</td><td>gi|73747879</td><td>Q9Y4E6</td><td>371</td><td>gi|21757082</td><td></td><td>420</td><td>gi|21928510</td><td>Q8TDU9</td><td>469</td><td>gi|14993776</td><td>Q9H1R3</td><td>518</td><td>gi|119631978</td><td></td></tr>
<tr><td>274</td><td>gi|50897852</td><td>Q3V6T2</td><td>323</td><td>gi|55957538</td><td>P51587</td><td>372</td><td>gi|42760220</td><td></td><td>421</td><td>gi|31415703</td><td>P07225</td><td>470</td><td>gi|156547039</td><td>Q9UK11</td><td>519</td><td>gi|11992190</td><td></td></tr>
<tr><td>275</td><td>gi|197115805</td><td></td><td>324</td><td>gi|46812315</td><td></td><td>373</td><td>gi|998395</td><td></td><td>422</td><td>gi|34364645</td><td>Q6N092</td><td>471</td><td>gi|17864094</td><td>Q96JP0</td><td>520</td><td>gi|1235783</td><td></td></tr>
<tr><td>276</td><td>gi|62871248</td><td></td><td>325</td><td>gi|66346676</td><td>Q96RL7</td><td>374</td><td>gi|14268412</td><td></td><td>423</td><td>gi|57222563</td><td>Q14008</td><td>472</td><td>gi|18041722</td><td></td><td>521</td><td>gi|125758</td><td>P01594</td></tr>
<tr><td>277</td><td>gi|4557691</td><td>P23276</td><td>326</td><td>gi|83305339</td><td>P11309</td><td>375</td><td>gi|42760062</td><td></td><td>424</td><td>gi|62897507</td><td>Q53GF8</td><td>473</td><td>gi|189053992</td><td>B2R9F2</td><td>522</td><td>gi|12733974</td><td></td></tr>
<tr><td>278</td><td>gi|84627489</td><td></td><td>327</td><td>gi|3142554</td><td></td><td>376</td><td>gi|58223262</td><td></td><td>425</td><td>gi|74146657</td><td></td><td>474</td><td>gi|189054394</td><td>B2RAM6</td><td>523</td><td>gi|1292863</td><td></td></tr>
<tr><td>279</td><td>gi|90823191</td><td></td><td>328</td><td>gi|33319606</td><td></td><td>377</td><td>gi|75707177</td><td></td><td>426</td><td>gi|74749396</td><td></td><td>475</td><td>gi|20379790</td><td>Q9Y5P3</td><td>524</td><td>gi|1335383</td><td></td></tr>
<tr><td>280</td><td>gi|62088774</td><td>P02452</td><td>329</td><td>gi|21753436</td><td>Q8N9I9</td><td>378</td><td>gi|149773468</td><td>Q09MP3</td><td>427</td><td>gi|92091575</td><td>Q2TAC2</td><td>476</td><td>gi|2114253</td><td>Q5NV68</td><td>525</td><td>gi|14043615</td><td></td></tr>
<tr><td>281</td><td>gi|422908</td><td>Q5NV65</td><td>330</td><td>gi|23683338</td><td></td><td>379</td><td>gi|4507353</td><td>Q92804</td><td>428</td><td>gi|110626504</td><td></td><td>477</td><td>gi|21669349</td><td></td><td>526</td><td>gi|145942180</td><td></td></tr>
<tr><td>282</td><td>gi|58202700</td><td></td><td>331</td><td>gi|119578374</td><td>P02458</td><td>380</td><td>gi|5051630</td><td>Q9Y6L6</td><td>429</td><td>gi|112699712</td><td></td><td>478</td><td>gi|21669395</td><td></td><td>527</td><td>gi|146336933</td><td>B1N7B8</td></tr>
<tr><td>283</td><td>gi|40388586</td><td></td><td>332</td><td>gi|2735715</td><td>P08123</td><td>381</td><td>gi|111917725</td><td></td><td>430</td><td>gi|114385755</td><td></td><td>479</td><td>gi|21669495</td><td></td><td>528</td><td>gi|152941042</td><td></td></tr>
<tr><td>284</td><td>gi|7021531</td><td>Q9UQD0</td><td>333</td><td>gi|6330422</td><td>Q9ULL8</td><td>382</td><td>gi|119588367</td><td>D3DQR0</td><td>431</td><td>gi|118406148</td><td></td><td>480</td><td>gi|22209001</td><td>Q96MW7</td><td>529</td><td>gi|153792678</td><td></td></tr>
<tr><td>285</td><td>gi|106897896</td><td></td><td>334</td><td>gi|4566035</td><td></td><td>383</td><td>gi|126539</td><td>P01700</td><td>432</td><td>gi|119601973</td><td>P08185</td><td>481</td><td>gi|27348189</td><td>Q8IXE7</td><td>530</td><td>gi|15723003</td><td></td></tr>
<tr><td>286</td><td>gi|3170943</td><td></td><td>335</td><td>gi|47846623</td><td></td><td>384</td><td>gi|145942614</td><td></td><td>433</td><td>gi|121941229</td><td>Q2KHM7</td><td>482</td><td>gi|3091196</td><td></td><td>531</td><td>gi|16117138</td><td></td></tr>
<tr><td>287</td><td>gi|58223162</td><td></td><td>336</td><td>gi|5802814</td><td></td><td>385</td><td>gi|170684552</td><td></td><td>434</td><td>gi|13376611</td><td>Q9H7S9</td><td>483</td><td>gi|33319340</td><td></td><td>532</td><td>gi|169179771</td><td></td></tr>
<tr><td>288</td><td>gi|34101286</td><td>Q96KR1</td><td>337</td><td>gi|84028237</td><td>Q9HB19</td><td>386</td><td>gi|2852417</td><td></td><td>435</td><td>gi|151302818</td><td></td><td>484</td><td>gi|37777990</td><td></td><td>533</td><td>gi|169206310</td><td></td></tr>
<tr><td>289</td><td>gi|62898904</td><td>Q53EQ3</td><td>338</td><td>gi|31657092</td><td>Q86UQ4</td><td>387</td><td>gi|3091180</td><td></td><td>436</td><td>gi|169169902</td><td></td><td>485</td><td>gi|425518</td><td></td><td>534</td><td>gi|170684398</td><td></td></tr>
<tr><td>290</td><td>gi|119583644</td><td>Q92794</td><td>339</td><td>gi|112695210</td><td></td><td>388</td><td>gi|41019505</td><td>Q04637</td><td>437</td><td>gi|17226636</td><td></td><td>486</td><td>gi|4572474</td><td>Q9Y250</td><td>535</td><td>gi|170684402</td><td></td></tr>
<tr><td>291</td><td>gi|13431717</td><td>Q9UKX3</td><td>340</td><td>gi|62897345</td><td>Q53GN8</td><td>389</td><td>gi|55957709</td><td>B1ALS2</td><td>438</td><td>gi|197115849</td><td></td><td>487</td><td>gi|47271320</td><td></td><td>536</td><td>gi|170684452</td><td></td></tr>
<tr><td>292</td><td>gi|18496791</td><td></td><td>341</td><td>gi|70798532</td><td></td><td>390</td><td>gi|57863250</td><td></td><td>439</td><td>gi|20521668</td><td>Q96T49</td><td>488</td><td>gi|49257462</td><td>Q6GMX3</td><td>537</td><td>gi|170684546</td><td></td></tr>
<tr><td>293</td><td>gi|86438950</td><td></td><td>342</td><td>gi|119583895</td><td>Q9NQT8</td><td>391</td><td>gi|66840148</td><td></td><td>440</td><td>gi|27261743</td><td></td><td>489</td><td>gi|51103397</td><td></td><td>538</td><td>gi|171853445</td><td></td></tr>
<tr><td>294</td><td>gi|112696257</td><td></td><td>343</td><td>gi|46254071</td><td></td><td>392</td><td>gi|18025706</td><td></td><td>441</td><td>gi|34527413</td><td></td><td>490</td><td>gi|56117850</td><td>O75534</td><td>539</td><td>gi|17226648</td><td></td></tr>
<tr><td>295</td><td>gi|119610295</td><td></td><td>344</td><td>gi|51259604</td><td></td><td>393</td><td>gi|24308336</td><td>Q5TGI0</td><td>442</td><td>gi|37777986</td><td></td><td>491</td><td>gi|58222796</td><td></td><td>540</td><td>gi|1813658</td><td></td></tr>
<tr><td>296</td><td>gi|9663172</td><td></td><td>345</td><td>gi|77158045</td><td></td><td>394</td><td>gi|2995691</td><td></td><td>443</td><td>gi|41152097</td><td>Q02040</td><td>492</td><td>gi|62089156</td><td>Q59EM6</td><td>541</td><td>gi|182639267</td><td>Q99797</td></tr>
<tr><td>297</td><td>gi|194391112</td><td>B4DPR2</td><td>346</td><td>gi|80975617</td><td></td><td>395</td><td>gi|33319586</td><td></td><td>444</td><td>gi|52545843</td><td>Q9Y597</td><td>493</td><td>gi|62861003</td><td></td><td>542</td><td>gi|1834497</td><td></td></tr>
<tr><td>298</td><td>gi|55962671</td><td>Q5SQ11</td><td>347</td><td>gi|21669319</td><td></td><td>396</td><td>gi|70798534</td><td></td><td>445</td><td>gi|5578790</td><td></td><td>494</td><td>gi|84040294</td><td>Q14690</td><td>543</td><td>gi|18698417</td><td></td></tr>
<tr><td>299</td><td>gi|9295281</td><td></td><td>348</td><td>gi|58222530</td><td></td><td>397</td><td>gi|91206450</td><td></td><td>446</td><td>gi|62898962</td><td>Q15542</td><td>495</td><td>gi|98956058</td><td></td><td>544</td><td>gi|190358761</td><td>A4D997</td></tr>
<tr><td>300</td><td>gi|106899620</td><td></td><td>349</td><td>gi|5921617</td><td>Q9UL82</td><td>398</td><td>gi|91979418</td><td></td><td>447</td><td>gi|6643219</td><td></td><td>496</td><td>gi|98956324</td><td></td><td>545</td><td>gi|1905799</td><td></td></tr>
<tr><td>301</td><td>gi|29837086</td><td></td><td>350</td><td>gi|112698094</td><td></td><td>399</td><td>gi|194385816</td><td></td><td>448</td><td>gi|68533041</td><td>Q567U6</td><td>497</td><td>gi|1071952</td><td></td><td>546</td><td>gi|194384564</td><td>B4DL80</td></tr>
<tr><td>302</td><td>gi|33318888</td><td></td><td>351</td><td>gi|169203189</td><td></td><td>400</td><td>gi|34364635</td><td>Q6N097</td><td>449</td><td>gi|7657554</td><td>Q9UK85</td><td>498</td><td>gi|109693283</td><td>Q0ZCI8</td><td>547</td><td>gi|197115583</td><td></td></tr>
<tr><td>303</td><td>gi|5419721</td><td></td><td>352</td><td>gi|21707884</td><td></td><td>401</td><td>gi|37747855</td><td></td><td>450</td><td>gi|81175178</td><td>P35527</td><td>499</td><td>gi|112694210</td><td></td><td>548</td><td>gi|21217667</td><td>Q8IWT0</td></tr>
<tr><td>304</td><td>gi|63054858</td><td>A6NED2</td><td>353</td><td>gi|34364807</td><td>Q6N030</td><td>402</td><td>gi|90823187</td><td></td><td>451</td><td>gi|112700674</td><td></td><td>500</td><td>gi|112701219</td><td></td><td>549</td><td>gi|21310450</td><td></td></tr>
<tr><td>305</td><td>gi|157427661</td><td>Q8N944</td><td>354</td><td>gi|93007497</td><td></td><td>403</td><td>gi|125815</td><td>P06311</td><td>452</td><td>gi|122892354</td><td></td><td>501</td><td>gi|114147463</td><td></td><td>550</td><td>gi|21362050</td><td>Q96NT0</td></tr>
<tr><td>306</td><td>gi|40788905</td><td>Q92598</td><td>355</td><td>gi|112700291</td><td></td><td>404</td><td>gi|1673595</td><td></td><td>453</td><td>gi|14589193</td><td></td><td>502</td><td>gi|114147640</td><td></td><td>551</td><td>gi|215982746</td><td></td></tr>
<tr><td>307</td><td>gi|46094081</td><td>Q9ULH1</td><td>356</td><td>gi|171854335</td><td></td><td>405</td><td>gi|51593790</td><td></td><td>454</td><td>gi|14602658</td><td></td><td>503</td><td>gi|114385588</td><td></td><td>552</td><td>gi|21619606</td><td>Q6PIL8</td></tr>
<tr><td>308</td><td>gi|46253780</td><td></td><td>357</td><td>gi|33319634</td><td></td><td>406</td><td>gi|74738655</td><td></td><td>455</td><td>gi|146330486</td><td></td><td>504</td><td>gi|114385734</td><td></td><td>553</td><td>gi|21668668</td><td></td></tr>
<tr><td>309</td><td>gi|116249459</td><td></td><td>358</td><td>gi|35825</td><td>P20742</td><td>407</td><td>gi|8927581</td><td>O75526</td><td>456</td><td>gi|15080499</td><td></td><td>505</td><td>gi|114385805</td><td></td><td>554</td><td>gi|21669539</td><td></td></tr>
<tr><td>310</td><td>gi|58222068</td><td></td><td>359</td><td>gi|453155</td><td></td><td>408</td><td>gi|10334595</td><td></td><td>457</td><td>gi|197115573</td><td></td><td>506</td><td>gi|118405869</td><td></td><td>555</td><td>gi|21998831</td><td></td></tr>
<tr><td>311</td><td>gi|112696057</td><td></td><td>360</td><td>gi|4103667</td><td></td><td>409</td><td>gi|112700812</td><td></td><td>458</td><td>gi|21620096</td><td>Q8N518</td><td>507</td><td>gi|118406312</td><td></td><td>556</td><td>gi|22095259</td><td></td></tr>
<tr><td>312</td><td>gi|194383688</td><td>B4DKJ0</td><td>361</td><td>gi|62531288</td><td>Q562E5</td><td>410</td><td>gi|123859</td><td>P01780</td><td>459</td><td>gi|22749359</td><td>Q9Y2I7</td><td>508</td><td>gi|118406366</td><td></td><td>557</td><td>gi|23194491</td><td></td></tr>
<tr><td>313</td><td>gi|47154912</td><td></td><td>362</td><td>gi|21751926</td><td>Q32P44</td><td>411</td><td>gi|158420731</td><td>Q12873</td><td>460</td><td>gi|34526178</td><td></td><td>509</td><td>gi|119571049</td><td></td><td>558</td><td>gi|2385496</td><td>A2IPI5</td></tr>
<tr><td>314</td><td>gi|112701089</td><td></td><td>363</td><td>gi|2765421</td><td></td><td>412</td><td>gi|21361959</td><td>Q8WV83</td><td>461</td><td>gi|4503625</td><td>P00742</td><td>510</td><td>gi|119573478</td><td></td><td>559</td><td>gi|2565063</td><td>Q9BT09</td></tr>
<tr><td>315</td><td>gi|116292750</td><td>P08519</td><td>364</td><td>gi|28273130</td><td>Q86YV0</td><td>413</td><td>gi|3093902</td><td></td><td>462</td><td>gi|55960308</td><td>Q96M02</td><td>511</td><td>gi|119584064</td><td>Q8N163</td><td>560</td><td>gi|29649727</td><td>Q86W19</td></tr>
<tr><td>316</td><td>gi|415821</td><td>P46013</td><td>365</td><td>gi|40788229</td><td>Q13615</td><td>414</td><td>gi|33318924</td><td></td><td>463</td><td>gi|58222833</td><td></td><td>512</td><td>gi|119607575</td><td>Q86Y56</td><td>561</td><td>gi|30173473</td><td>Q96T25</td></tr>
<tr><td>317</td><td>gi|18025666</td><td></td><td>366</td><td>gi|4324078</td><td></td><td>415</td><td>gi|3980130</td><td>Q9NSD0</td><td>464</td><td>gi|6013427</td><td></td><td>513</td><td>gi|119617920</td><td></td><td>562</td><td>gi|30722448</td><td>Q86T24</td></tr>
<tr><td>318</td><td>gi|18025646</td><td></td><td>367</td><td>gi|57864582</td><td>Q86YZ3</td><td>416</td><td>gi|39938169</td><td></td><td>465</td><td>gi|94574438</td><td>Q1JQ81</td><td>514</td><td>gi|119622859</td><td></td><td>563</td><td>gi|30841940</td><td></td></tr>
<tr><td>319</td><td>gi|3273353</td><td></td><td>368</td><td>gi|58221609</td><td></td><td>417</td><td>gi|70798697</td><td></td><td>466</td><td>gi|112695007</td><td></td><td>515</td><td>gi|119623046</td><td></td><td>564</td><td>gi|3093872</td><td></td></tr>
</table>

565	gi|31337686		614	gi|576554		663	gi|7020538	Q53EW4	712	gi|190410954	A8MYB1	761	gi|33569996		810	gi|85542144	Q562F6
566	gi|31377611		615	gi|58222425		664	gi|50513245	Q13111	713	gi|193786724	B3KQI5	762	gi|41393547	A2RRP1	811	gi|33354053	A4LAA3
567	gi|3142600		616	gi|58222604		665	gi|486879		714	gi|194376932	B4DWG5	763	gi|11121491		812	gi|37780053	Q2TV78
568	gi|31542733	Q8N2U9	617	gi|58223154		666	gi|17223624	Q8IUA7	715	gi|19773365		764	gi|115511046	O95789	813	gi|194385282	B4E256
569	gi|32440849	Q2Z197	618	gi|587249		667	gi|39794204	Q5VV67	716	gi|21928977	Q8NGF2	765	gi|169216868		814	gi|47154910	
570	gi|33235632		619	gi|6179862		668	gi|118764321	P82987	717	gi|31338739		766	gi|189053887	B2R8F9	815	gi|194379064	B4DHC2
571	gi|33319140		620	gi|6179870		669	gi|37694647		718	gi|39938022		767	gi|50949507	Q69YJ7	816	gi|47939097	Q6IQ08
572	gi|33319290		621	gi|62088088	P02786	670	gi|52317182	Q8NGN2	719	gi|4261843		768	gi|119607051		817	gi|5453810	O00634
573	gi|33319342		622	gi|62113341	Q56G89	671	gi|1526978	Q92736	720	gi|438069	P32119	769	gi|15530238	Q9H9L4	818	gi|112695992	
574	gi|33319510		623	gi|62720451		672	gi|5326898	Q9Y5B0	721	gi|4506325	Q15256	770	gi|16550719		819	gi|114386054	
575	gi|33319544		624	gi|62822092		673	gi|54633198	P10586	722	gi|46397468	Q14533	771	gi|21669417		820	gi|119580386	A8K8P3
576	gi|33319576		625	gi|62860986		674	gi|55662488	Q5VZ84	723	gi|51103413		772	gi|266666	P30531	821	gi|194379192	B4DHI5
577	gi|33570097		626	gi|62861025		675	gi|119591496	Q86XH1	724	gi|56204710	Q9NZB8	773	gi|34303926	Q7L3T8	822	gi|45243534	Q9HC56
578	gi|34526220		627	gi|62897339	Q53GP1	676	gi|18089152	Q9Y295	725	gi|57242761	Q13546	774	gi|587305		823	gi|46411193	Q2TU65
579	gi|3608459		628	gi|63103065		677	gi|55743104		726	gi|587267		775	gi|6642738	Q9H8H3	824	gi|4837755	
580	gi|3721651		629	gi|64654113	Q92832	678	gi|119600834		727	gi|62088564	Q59FG8	776	gi|66529294	Q15165	825	gi|50949980	O75153
581	gi|3954956		630	gi|6643391		679	gi|119629733		728	gi|62897057	Q99933	777	gi|7019933	Q9NXS9	826	gi|62089272	Q59EG8
582	gi|39930571	Q8IYG6	631	gi|66821507		680	gi|57208525	B3KTY4	729	gi|67514036	Q5JPT5	778	gi|10439721	Q9H5R7	827	gi|112696443	
583	gi|39938092		632	gi|66821561		681	gi|62089310	Q59EE9	730	gi|70798576		779	gi|106897473		828	gi|11493431	Q9H3B0
584	gi|39938127		633	gi|70798688		682	gi|112695358		731	gi|75707569		780	gi|112699453		829	gi|119591050	Q96BH1
585	gi|39938204		634	gi|70798801		683	gi|119623194		732	gi|7702778	P59666	781	gi|112701481		830	gi|119614919	
586	gi|40807038	Q6P163	635	gi|70888243		684	gi|28269794	Q96PQ8	733	gi|8118530	Q9Y371	782	gi|11612390	Q9H2S9	831	gi|119622987	
587	gi|4321847		636	gi|74147093		685	gi|6635135	Q8N6R0	734	gi|89477404	Q1A5X7	783	gi|119597150	B3KSQ0	832	gi|169169217	
588	gi|4323179		637	gi|75707473		686	gi|169202103		735	gi|169212806		784	gi|14716960		833	gi|2306828	
589	gi|4378388		638	gi|76252629		687	gi|36029914	Q96JH7	736	gi|68533111	Q4LE43	785	gi|16751921	P81605	834	gi|28637	Q13747
590	gi|4503011	P15169	639	gi|77378306		688	gi|76789661	Q9UL68	737	gi|98956178		786	gi|169165187		835	gi|33319050	
591	gi|4505735	P10720	640	gi|77379751		689	gi|8810056		738	gi|12585192	Q9NR09	787	gi|169167744		836	gi|33868637	
592	gi|4505949	P01189	641	gi|7959791		690	gi|10439979	Q9Y2T2	739	gi|6469374	Q9NY15	788	gi|170684480		837	gi|38372737	Q8NGN7
593	gi|45439365	Q14CX5	642	gi|80975581		691	gi|194385510	B4E2H0	740	gi|106640		789	gi|194381382	Q8N4V1	838	gi|55957576	Q8TC20
594	gi|460857		643	gi|83405168	Q2TAM5	692	gi|4505541	O60763	741	gi|27348193	Q8IXE5	790	gi|31337528		839	gi|58198523	
595	gi|46254012		644	gi|9295291		693	gi|58257654	Q5T2D3	742	gi|50355941	Q86SQ4	791	gi|3142454		840	gi|6330847	Q9UN36
596	gi|47846717		645	gi|98956018		694	gi|169218213		743	gi|71296783	Q49AP2	792	gi|42760084		841	gi|7023653	Q9BZD2
597	gi|480866		646	gi|98956342		695	gi|18203332	Q9NZN3	744	gi|84798160		793	gi|4503109	P01036	842	gi|7959193	Q9P262
598	gi|480919		647	gi|119592748		696	gi|194379290	B4DY49	745	gi|194374471	B4DEM0	794	gi|51949939		843	gi|82792219	Q2VCE3
599	gi|481342		648	gi|15277841	Q9NP71	697	gi|4826762	P00738	746	gi|8886722	Q9NRR4	795	gi|52317094	Q8IXE1	844	gi|89242141	Q96DM3
600	gi|49065410	Q6FID4	649	gi|169163482		698	gi|73760405	P42167	747	gi|4758416	Q92538	796	gi|6649884		845	gi|98956298	
601	gi|49523902		650	gi|169217275		699	gi|112696917		748	gi|68051186	Q4R9M7	797	gi|66932890		846	gi|16974104	
602	gi|5051143		651	gi|7022973	Q8WWK9	700	gi|113205085	Q6UY01	749	gi|94721336	Q96T51	798	gi|68533117	Q4LE40	847	gi|4098868	Q9UQB3
603	gi|5081713		652	gi|189066649	Q96P44	701	gi|114385630		750	gi|119597857	Q9UES4	799	gi|71297396	Q499Z2	848	gi|15777233	
604	gi|51095056	A4D112	653	gi|9663254		702	gi|118406112		751	gi|14165419	Q9UN72	800	gi|73760409	P10646	849	gi|21670677	
605	gi|51103541		654	gi|194386662	B4DR29	703	gi|119588117		752	gi|90101808	P11171	801	gi|7657647	Q9NZR1	850	gi|119605583	
606	gi|51490957		655	gi|189046909	A1L4H1	704	gi|119624788		753	gi|52545703	Q9BXF3	802	gi|81174549	Q7Z4H9	851	gi|21706895	
607	gi|54038911		656	gi|194383506	B4E1B2	705	gi|119625347	Q6V1P9	754	gi|71891685	Q86VP6	803	gi|98956256		852	gi|28912912	Q9C056
608	gi|54781103		657	gi|1008070		706	gi|12054076		755	gi|46249406		804	gi|98956415		853	gi|49257892	Q6GMV7
609	gi|5578810		658	gi|119578529	Q96GM5	707	gi|121942687	Q3LI62	756	gi|7243169	A5YM72	805	gi|21669475		854	gi|55957320	Q76FK4
610	gi|55859590	Q96IY4	659	gi|22761758	Q17RM4	708	gi|12750761		757	gi|119620894	B3KV89	806	gi|28278130	Q86XF2	855	gi|5689776	Q9UMQ6
611	gi|55957432	Q5JVP3	660	gi|57864228	Q92952	709	gi|13376439	Q9HAC8	758	gi|40846989	Q6RR68	807	gi|31335203		856	gi|62898976	Q8IU81
612	gi|56204877	Q5TB12	661	gi|8843933	Q9NRR5	710	gi|145942603		759	gi|118150678	Q008S8	808	gi|62087782	Q59GK8	857	gi|65507253	
613	gi|57208830	Q5JVE8	662	gi|154090979	Q9ULL0	711	gi|169213473		760	gi|194374401	B4DEI5	809	gi|64464726	Q14678	858	gi|7020071	Q8IUZ0

859	gi|77158065		908		P23083	957		Q9NYF0	1006		Q8IXB1	1055		P78426	1104		A6NNW6
860	gi|118425776		909		P01714	958		O95714	1007		P06681	1056		P83593	1105		O43613
861	gi|119575152		910		P01771	959		P01768	1008		Q12860	1057		Q0P6D2	1106		O43763
862	gi|119617692		911		P04259	960		O94833	1009		O95071	1058		Q12766	1107		O95453
863	gi|14210488	Q9BTE1	912		P06316	961		Q9UKG1	1010		P02788	1059		Q13574	1108		P04211
864	gi|189041198	Q5T699	913		P48668	962		Q9BTC0	1011		P04434	1060		Q14914	1109		P04438
865	gi|189054552		914		P01613	963		A6NCL2	1012		P24043	1061		Q4G0U5	1110		P21283
866	gi|22748713	Q96BM1	915		P01765	964		P01769	1013		P49366	1062		Q6UXN2	1111		P30260
867	gi|338733		916		P02750	965		Q5JSZ5	1014		Q13144	1063		Q6ZTN6	1112		Q15131
868	gi|4506577	P35398	917		P06309	966		Q9ULL1	1015		Q4VC44	1064		Q7L0R7	1113		Q16620
869	gi|542885		918		P19652	967		P01709	1016		Q7Z4L5	1065		Q8WWC4	1114		Q3LI73
870	gi|58223072		919		P23142	968		P01760	1017		Q9Y238	1066		Q9BSK4	1115		Q460N5
871	gi|62897259	Q53GT1	920		P51884	969		P20929	1018		Q9H2G4	1067		Q9GZU7	1116		Q5VVJ2
872	gi|6760045	Q9H320	921		P69891	970		P58107	1019		Q9UDX5	1068		Q9H079	1117		Q6P2C8
873	gi|70798113		922		P01602	971		Q9Y4D7	1020		O15234	1069		Q9UHQ7	1118		Q86YR7
874		P01857	923		P01824	972		P01615	1021		P48549	1070		Q9Y5F1	1119		Q8IU85
875		P0C0L5	924		P01825	973		P04209	1022		P80421	1071		Q9BV73	1120		Q8WV41
876		P04114	925		P06326	974		Q05707	1023		Q9BXT4	1072		Q8IVF4	1121		Q92766
877		P01834	926		P01625	975		Q14151	1024		Q96J65	1073		Q00975	1122		Q96SE7
878		P0C0L4	927		P01716	976		Q07869	1025		O75159	1074		Q9UBZ9	1123		Q9H074
879		P01842	928		P01617	977		Q9BYW2	1026		P01699	1075		Q5VUJ6	1124		Q9NTZ6
880		P00751	929		P04430	978		P01767	1027		P29375	1076		Q5T9A4	1125		Q9NVH1
881		P01860	930		P35542	979		Q8NE71	1028		P56179	1077		Q15596	1126		Q9UKN7
882		P01620	931		P01622	980		P01612	1029		P82979	1078		Q6P0Q8	1127		Q9UPX8
883		P04206	932		P06310	981		Q86VF7	1030		Q01101	1079		Q92771	1128		Q9Y5J6
884		P18136	933		P80419	982		O95785	1031		Q16352	1080		Q9HCK8	1129		P52746
885		P01764	934		P33151	983		Q17RD7	1032		Q5MIZ7	1081		Q6PKG0	1130		Q8IXT5
886		P01861	935		P26927	984		A6NE01	1033		Q86Z23	1082		Q9GZR1	1131		O43298
887		P01019	936		P01770	985		P00395	1034		Q8N3Y3	1083		Q4G0P3	1132		Q96L96
888		P01614	937		P01621	986		P01777	1035		Q9HCD5	1084		Q92581	1133		Q8TBZ5
889		P68871	938		Q96NX5	987		P02776	1036		Q9UHQ9	1085		Q9P296	1134		Q5JSJ4
890		P04196	939		P01773	988		P63128	1037		Q9UN76	1086		Q9UI47	1135		Q8NG97
891		P06314	940		P01719	989		Q69YH5	1038		O15481	1087		Q2M3M2	1136		Q6NSI4
892		Q08380	941		P01593	990		Q9NXA8	1039		P01598	1088		Q68DA7	1137		P80192
893		P80748	942		P01619	991		P23193	1040		P01605	1089		Q9BZK7	1138		Q9BZL4
894		P04207	943		P01703	992		Q9NW08	1041		P01611	1090		O95425	1139		O95359
895		P06312	944		P01708	993		Q6P2P2	1042		P01701	1091		P78332	1140		Q8TD84
896		P01772	945		P02655	994		Q5BJE1	1043		P01721	1092		P98198	1141		Q9UGM6
897		P02775	946		P06319	995		Q8IYA6	1044		P01775	1093		Q8NDH2	1142		O14531
898		P04433	947		P01624	996		Q9Y6K5	1045		P01779	1094		A6H8Y1	1143		P38435
899		P01608	948		P06887	997		P25940	1046		P02545	1095		Q9H0B6	1144		Q92794
900		P01717	949		P00736	998		Q8TEK3	1047		P06317	1096		B2RTY4	1145		Q9NX07
901		P01781	950		Q02224	999		P23467	1048		P06318	1097		O15018	1146		A6NCI4
902		P01766	951		P01704	1000		Q5TAX3	1049		P06889	1098		P51857	1147		B1ANY3
903		P04208	952		P41222	1001		O15027	1050		P07196	1099		Q13148	1148		P48048
904		P05156	953		Q15751	1002		Q86UR5	1051		P07686	1100		Q13823	1149		P50213
905		P06313	954		Q9Y2W1	1003		P01778	1052		P08575	1101		Q32P28	1150		Q5JTD7
906		P01601	955		P01762	1004		Q29980	1053		P17020	1102		Q9P2D0	1151		Q5T5M9
907		P01743	956		Q92793	1005		Q8IYR0	1054		P18065	1103		A2RUS2	1152		Q96NJ5

1153		Q9HCM1	1164		Q9UPV0	1175		A6NJ16	1186		Q3ZCV2	1197		Q6IEE7	1208		Q96PV6
1154		A6NKC9	1165		A6NIX2	1176		O15409	1187		Q6ZV70	1198		O60245	1209		Q9BXW9
1155		Q6ZVL6	1166		Q92618	1177		O43353	1188		Q86YW5	1199		A6H8M9	1210		Q9NVS9
1156		Q8IZU1	1167		Q12770	1178		P01610	1189		Q8NHP7	1200		Q13496			
1157		Q96PF1	1168		Q49AM3	1179		P01742	1190		Q9H013	1201		Q8IVS2			
1158		Q9BYV8	1169		O60333	1180		P01918	1191		Q9H165	1202		Q8N2U0			
1159		Q9H8S5	1170		P01763	1181		P09067	1192		Q9NWT6	1203		Q92562			
1160		Q9NRK6	1171		P08572	1182		P31942	1193		Q9UDY8	1204		Q96QF7			
1161		Q9H2X9	1172		Q14739	1183		P46781	1194		P51825	1205		Q9NZP6			
1162		Q8TDM6	1173		Q6P4E1	1184		Q13530	1195		P42224	1206		O43182			
1163		P19174	1174		Q96FV2	1185		Q13641	1196		Q5T5P2	1207		Q8N7X4			

Supplemental Material, Table 2. List of FDA-approved biomarker proteins for adult plasma analytics (Anderson 2010) that also were observed in this study in UCB serum.

UniProt Accession	Protein name brief	Protein Name	CPT from Ref. labs*	KEGG pathway number
P02768	ALBU	Albumin	82040	
P02763	A1AG1	Alpha-1-Acid Glycoprotein (Orosomucoid)	82985	
P19652	A1AG2	Alpha-1-Acid Glycoprotein (Orosomucoid)	82985	
P01009	SERPINA1	Alpha-1-Antitrypsin	82103	04610
P08697	SERPINF2	Alpha-2-Antiplasmin	85410	04610
P02765	FETUA	Alpha-2-HS-Glycoprotein		
P01023	A2M	Alpha-2-Macroglobulin	83883	04610
P02771	FETA	Alpha-Fetoprotein - Tumor Marker	82105	
P01008	SERPINC1	Antithrombin III	85301	04610
P02647	APOA1	Apolipoprotein A1	82172	03320
P04114	APOB	Apolipoprotein B	82172	
P02775	PPBP	Beta-Thromboglobulin		04062, 04060
P00450	CP	Ceruloplasmin	82390	00860
P09871	C1S	Complement C1	86161	04610, 05322, 05150
P00736	C1R	Complement C1	86161	04610, 05322, 05150
P05155	SERPING1	Complement C1 Inhibitor	86160	04610
P01024	C3	Complement C3	86160	04610, 05322, 05150
P0C0L4	C4A	Complement C4	86160	04610, 05322, 05150
P01031	C5	Complement C5	86160	04610, 05322, 05150
P01034	CYTC	Cystatin C	82610	04970
P00742	F10	Factor X	85260	04610
Q96PQ8	F7	Factor VII	85230	04610
P05160	F13B	Factor XIII	85290	04610
P02671	FIBA	Fibrinogen	85385	04610
P02751	FN1	Fibronectin		05100, 04810, 05200, 04512, 04510, 05146
P00738		Haptoglobin	83010	
P02790	HEMO	Hemopexin		
Q5EK51		Lactoferrin		
P08519	APOA	Lipoprotein(a) (Lp(a))	83695	
P00747	PLG	Plasminogen	85421	04610, 05150, 04080
P02766	TTHY	Prealbumin	84134	
P00751	CFB	Properdin factor B	83883	04610, 05150
P07225	PROS1	Protein S	85305	04610
P02753	RET4	Retinol binding protein (RBP)	83883	
P05543	THBG	Thyroxine Binding Globulin (TBG)	84442	
P02787	TRFE	Transferrin	84466	
P02786	TFRC	Transferrin Receptor (TFR)	84238	04144, 04640, 04145
P04275	VWF	Von Willebrand Factor	85246	04610, 04512, 04510

* CPT were extracted.

References

Anderson N.L. 2010. The clinical plasma proteome: a survey of clinical assays for proteins in plasma and serum. Clin Chem 56:177-185.
